# A taxonomic study of *Nemania* from China, with six new species

**DOI:** 10.3897/mycokeys.83.69906

**Published:** 2021-08-24

**Authors:** Yin Hui Pi, Si Han Long, You Peng Wu, Li Li Liu, Yan Lin, Qing De Long, Ji Chuan Kang, Ying Qian Kang, Chu Rui Chang, Xiang Chun Shen, Nalin N. Wijayawardene, Xu Zhang, Qi Rui Li

**Affiliations:** 1 State Key Laboratory of Functions and Applications of Medicinal Plants, Guizhou Medical University, Guiyang 550004, China Guizhou Medical University Guiyang China; 2 The High Efficacy Application of Natural Medicinal Resources Engineering Center of Guizhou Province (The Key Laboratory of Optimal Utilization of Natural Medicine Resources), School of Pharmaceutical Sciences, Guizhou Medical University, University Town, Guian New District, Guizhou, China Guizhou University Guiyang China; 3 Engineering and Research Center for Southwest Bio-Pharmaceutical Resources of National Education Ministry of China, Guizhou University, Guiyang, Guizhou 550025, China Qujing Normal University Qujing China; 4 Key Laboratory of Environmental Pollution Monitoring and Disease Control, Ministry of Education of Guizhou and Guizhou Talent Base for Microbiology and Human Health, School of Basic Medical Sciences, Guizhou Medical University, Guiyang, China Guizhou Medical University Guiyang China; 5 Center for Yunnan Plateau Biological Resources Protection and Utilization, College of Biological Resource and Food Engineering, Qujing Normal University, Qujing, Yunnan 655011, China Guizhou University Guiyang China; 6 Section of Genetics, Institute for Research and Development in Health and Social Care, No: 393/3, Lily Avenue, Off Robert Gunawardane Mawatha, Battaramulla 10120, Sri Lanka Qujing Normal University Qujing China

**Keywords:** phylogeny, six new species, taxonomy, Xylariaceae

## Abstract

During an investigation of Xylariaceae from 2019 to 2020, isolates representing eight *Nemania* (Xylariacese) species were collected from Yunnan, Guizhou and Hainan Provinces in China. Morphological and multi-gene phylogenetic analyses, based on combined ITS, α-actin, *rpb2* and β-tubulin sequences, confirmed that six of them are new to science, viz. *Nemaniacamelliae*, *N.changningensis*, *N.cyclobalanopsina*, *N.feicuiensis*, *N.lishuicola* and *N.rubi*; one is a new record (*N.caries*) for China and one is a known species (*N.diffusa*). Morphological descriptions and illustrations of all species are detailed. In addition, the characteristics of *Nemania* are summarised and prevailing contradictions in generic concepts are discussed.

## Introduction

*Nemania* Gray was established by Gray (1821) for a heterogeneous assemblage of taxa and was affiliated with *Xylariaceae* Tul. & C. Tul. Since the early taxonomic description of this genus was ambiguous, taxonomists have often regarded some species of *Nemania* as synonyms of *Hypoxylon* Bull. For example, *Nemaniaangusta* (Petch) Y.M. Ju & J. D. Rogers was regarded as a synonym of *Hypoxylonangustum* Petch. (Miller 1961; Whalley et al. 1983; Ju and Rogers 2002). Subsequently, the generic concept of *Nemania* was modified by Pouzar (1985a, b) and Petrini and Rogers (1986). Granmo et al. (1999) and Ju and Rogers (2002) provided a comprehensive background to *Nemania* and accepted 37 species. Sánchez-Ballesteros et al. (2000) used the internal transcribed spacers (ITS) sequence to perform a phylogenetic study of *Nemania*, which supported the segregation of *Nemania* from *Hypoxylon*. However, their conclusion was based only on ITS sequences and *Xylaria* Hill & Schrank was not included in this study. Hence, the generic placement of *Nemania* in the *Xylariaceae* was unclear. Hsieh et al. (2005) used β-tubulin and α-actin to evaluate the phylogenetic relationship of several xylariaceous genera. It was found to be particularly useful in xylariaceous fungias limited success in using ribosomal DNA genes to delineating genera and resolving generic relationships (Tang et al. 2007). Tang et al. (2007) re-established the phylogenetic relationships of *Nemania* with related genera, based on the combined dataset of ITS and *rpb2* which supported the separation of *Nemania* from *Hypoxylon*. However, Tang et al. (2007) stated that *Nemania* is closely related to *Xylaria* and phylogenetically distinct from *Annulohypoxylon* Y.M. Ju et al., *Daldinia* Ces. & De Not. and *Hypoxylon*. Ultimately, the boundaries of the genus became relatively clear and *Nemania* has been accepted as a distinct genus in *Xylariaceae* (Ju and Rogers 2002). The major morphological characteristics of *Nemania* include dark brown to black stromata, carbonaceous or at least brittle and not yielding pigments in 10% potassium hydroxide (KOH) (Ju and Rogers 2002), white soft tissue existing between or below the perithecia, ascospores usually pale brown and most of them have no obvious germ-slit and spore dehiscence in 10% KOH (Tang et al. 2007).

*Nemania* accepted 37 species by 2002, which occurs mainly distributed on the rotting wood of angiosperms (Ju and Rogers 2002; Tang et al. 2007). There are a few species introduced from China in recent years. Two new species (*N.flavitextura* Y.M. Ju, H.M. Hsieh & J.D. Rogers and *N.primolutea* Y.M. Ju, H.M. Hsieh & J.D. Rogers), collected from Taiwan, were reported by Ju et al. (2005). One new species and two new record species were discovered and described by Du et al. (2016) and Ariyawansa et al. (2015) in China. Recently, two new species (*N.yunnanensis* Tibpromma & Lu and *N.aquilariae* Tibpromma & Lu), collected from Yunnan Province, China, were discovered by Tibpromma et al. (2021). Ninety-three epithets of *Nemania* are listed on Index Fungorum (2021) (accession date: 06. 2021). Only 17 species of *Nemania* with gene sequences were retrieved from the NCBI database (https://www.ncbi.nlm.nih.gov) and morphological methods are the main distinguishing method for *Nemania*. Morphologically, it is mainly distinguished according to the germ slit, the size of the ascospores and the characteristics of the stromata.

In this study, eight species of *Nemania*, collected from Guizhou, Hainan and Yunnan Provinces in China, are introduced. Six new species are identified, based on morpho-molecular analyses, while *N.caries* is reported as a new record for China; *N.diffusa* has been previously reported from China (Du 2015). Detailed morphological descriptions, illustrations and phylogenetic information of all species are provided in this paper.

## Materials and methods

### Collection, isolation and morphology

Samples of rotting wood with fungiwere collected from October 2019 to December 2020 in various nature reserves of Guizhou, Hainan and Yunnan Provinces, China. These samples were placed in sealed bags and the coordinates of sampling sites (such as latitude, longitude and altitude) were recorded. Specimens were taken to the laboratory for examination. Microscopic observations were made with fungimounted in distilled water. A drop of Melzer’s Reagent was added to determine whether or not the ascus apical ring blued (the amyloid iodine reaction) and the reaction and morphology of the ring could be observed. Fragments of stroma and perithecial wall were placed in 10% KOH on a microscope slide and the extractable pigment observed. Pure cultures were obtained with the single spore isolation method (Long et al. 2019) and the cultures were grown on oatmeal agar (OA) and potato dextrose agar (PDA).

Morphological examination of fungion the rotting wood followed the methods of Xie et al. (2020). The characteristics of the stromata were observed with an Olympus SZ61 stereomicroscope and photographed using a fitted Canon 700D digital camera. The photomicrographs of asci and ascospores were taken with a Nikon digital camera (700D) fitted to a light microscope (Nikon Ni). Adobe Photoshop CS6 was used to arrange all the microphotographs. Measurements were performed using the Tarosoft image framework (v. 0.9.0.7). At least 30 ascospores, asci and ascus apical apparatus were measured for each specimen.

To prepare herbarium materials, the colonies grown on PDA were transferred to three 1.5 ml microcentrifuge tubes filled with sterile water and stored at 4 °C or with 10% glycerol at –20 °C. Herbarium materials were deposited in the Herbarium of Guizhou Medical University (**GMB**) and Herbarium of Kunming Institute of Botany, Chinese Academy of Sciences (**KUN**). Living cultures were deposited at Guizhou Medical University Culture Collection (**GMBC**).

### DNA extraction, PCR amplification and sequencing

The BIOMIGA Fungal Genomic DNA Extraction Kit (GD2416, Biomiga, USA) was used to extract genomic DNA from fresh fungal mycelium, according to the manufacturer’s instructions. The extracted DNA was stored at –20 °C.

Target regions of internal transcribed spacers (ITS) and RNA polymerase II second largest subunit (*rpb2*) regions were amplified symmetrically using primers of ITS4/ITS5 (White et al. 1990; Gardes and Bruns 1993) and fRPB2-5F/fRPB2-7cR (Liu et al. 1999), respectively. ACT512F and ACT783R (Hsieh et al. 2005) and T11 and T22 (Tanaka et al. 2009; Hsieh et al. 2010) primers were used for the amplification of the α-actin gene (ACT) and β-tubulin (TUB2), respectively. The components of the polymerase chain reaction (PCR) mixture and thermal cycling programme were performed as described by Pi et al. (2020). The amplified PCR fragments were sent to Sangon Biotech (Shanghai) Co., China, for sequencing. All newly-generated sequences of ITS, α-actin, *rpb2* and β-tubulin regions were uploaded to the GenBank database and the accession numbers are shown in Table 1.

**Table 1.** Taxa of *Nemania* and related genera used for phylogenetic analyses and their GenBank accession numbers.

Species	Strain number	GenBank Accession number				References
		ITS	*rpb2*	β-tubulin	α-actin	
* Amphirosellinia fushanensis *	HAST 91111209 (HT)	GU339496	GQ848339	GQ495950	GQ452360	Hsieh et al. (2010)
* Am. nigrospora *	HAST 91092308 (HT)	GU322457	GQ848340	GQ495951	GQ452361	Hsieh et al. (2010)
* Astrocystis bambusae *	HAST 89021904	GU322449	GQ844836	GQ495942	GQ449239	Hsieh et al. (2010)
* As. bambusicola *	MFLUCC 17-0127 (HT)	MF467942	MF467946	N/A	N/A	Hyde et al. (2017)
* As. concavispora *	MFLUCC 14-0174	KP297404	KP340532	KP406615	N/A	Daranagama et al. (2015)
* As. mirabilis *	HAST 94070803	GU322448	GQ844835	GQ495941	GQ449238	Hsieh et al. (2010)
* Brunneiperidium gracilentum *	MFLUCC 14-0011 (HT)	KP297400	KP340528	KP406611	N/A	Daranagama et al. (2015)
* B. involucratum *	MFLUCC 14-0009	KP297399	KP340527	KP406610	N/A	Daranagama et al. (2015)
* Collodiscula bambusae *	GZUH0102	KP054279	KP276675	KP276674	N/A	Li et al. (2015b)
* C. fangjingshanensis *	GZUH0109 (HT)	KR002590	KR002592	KR002589	N/A	Li et al. (2015a)
* C. leigongshanensis *	GZUH0107 (HT)	KP054281	KR002588	KR002587	N/A	Li et al. (2015a)
* C. tubulosa *	GACP QR0111 (HT)	MN017302	MN018403	MN018405	MN018402	Xie et al. (2020)
* Daldinia bambusicola *	CBS 122872 (HT)	KY610385	KY624241	AY951688	KU684037	Hsieh et al. (2005), Wendt et al. (2018)
* Dematophora buxi *	JDR 99	GU300070	GQ844780	GQ470228	GQ398228	Hsieh et al. (2010)
* De. necatrix *	CBS 349.36	AY909001	KY624275	KY624310	N/A	Pelaez et al. (2008), Wendt et al. (2018)
* Discoxylaria myrmecophila *	JDR 169	GU322433	GQ844819	GQ487710	GQ438747	Hsieh et al. (2010)
* Entoleuca mammata *	JDR 100	GU300072	GQ844782	GQ470230	GQ398230	Hsieh et al. (2010)
* Hypoxylon pulicicidum *	CBS 122622 (HT)	JX183075	KY624280	JX183072	JX183071	Bills et al. (2012), Wendt et al. (2018)
* Kretzschmariella culmorum *	JDR 88	KX430043	KX430045	KX430046	KX430044	Johnston et al. (2016)
* Nemania abortiva *	BISH 467 (HT)	GU292816	GQ844768	GQ470219	GQ374123	Hsieh et al. (2010)
* N. aenea *	CBS 680.86	AJ390427	N/A	N/A	N/A	Tang et al. (2007)
N. aenea var. aureolutea	ATCC 60819	AJ390428	N/A	N/A	N/A	Tang et al. (2007)
* N. aquilariae *	KUMCC 20-0268 (HT)	MW729422	MW717891	MW881142	MW717889	Tibpromma et al. (2021)
* N. beaumontii *	HAST 405	GU292819	GQ844772	GQ470222	GQ389694	Wendt et al. (2018)
* N. bipapillata *	HAST 90080610	GU292818	GQ844771	GQ470221	GQ389693	Hsieh et al. (2010)
* N. camelliae *	GMB0067	MW851888	MW836056	MW836030	MW836047	This study
	GMB0068 (HT)	MW851889	MW836055	MW836029	MW836046	This study
* N. caries *	GMB0069	MW851873	MW836069	MW836035	MW836051	This study
	GMB0070	MW851874	MW836071	MW836036	MW836050	This study
* N. changningensis *	GMB0056 (HT)	MW851875	MW836061	MW836027	MW836042	This study
	GMB0057	MW851876	MW836062	MW836028	MW836043	This study
* N. chestersii *	JF 04024	AJ390430	DQ631949	DQ840089	N/A	Tang et al. (2007, 2009)
* N. cyclobalanopsina *	GMB0061	MW851882	MW836058	MW836026	MW836039	This study
	GMB0062 (HT)	MW851883	MW836051	MW836025	MW836038	This study
* N. diffusa *	HAST 91020401	GU292817	GQ844769	GQ470220	GQ389692	Hsieh et al. (2010)
	GMB0071	MW851877	MW836067	MW836031	MW836053	This study
	GMB0072	MW851878	MW836068	MW836032	MW836052	This study
* N. feicuiensis *	GMB0058	MW851879	MW836064	MW836024	MW836045	This study
	GMB0059 (HT)	MW851880	MW836063	MW836023	MW836044	This study
* N. fusoidispora *	GZUH0098	MW851881	MW836070	MW836037	MW836054	Ariyawansa et al. (2015)
* N. illita *	YMJ 236	EF026122	GQ844770	EF025608	EF025593	Hsieh et al. (2010)
* N. rubi *	GMB0063	MW851884	MW836060	MW836022	MW836041	This study
	GMB0064 (HT)	MW851885	MW836059	MW836021	MW836040	This study
* N. lishuicola *	GMB0065 (HT)	MW851886	MW836065	MW836033	MW836048	This study
	GMB0066	MW851887	MW836066	MW836034	MW836049	This study
* N. macrocarpa *	WSP 265	GU292823	GQ844776	GQ470226	GQ389698	Hsieh et al. (2010)
* N. maritima *	HAST 89120401 (ET)	GU292822	GQ844775	GQ470225	GQ389697	Hsieh et al. (2010), Li et al. (2015a, b)
* N. plumbea *	JF TH-04-01	DQ641634	DQ631952	DQ840084	N/A	Tang et al. (2007, 2009)
* N. primolutea *	YMJ 91102001 (HT)	EF026121	GQ844767	EF025607	EF025592	Hsieh et al. (2010)
* N. serpens *	HAST 235	GU292820	GQ844773	GQ470223	GQ389695	Hsieh et al. (2010), Li et al. (2015a, b)
* N. sphaeriostoma *	JDR 261	GU292821	GQ844774	GQ470224	GQ389696	Hsieh et al. (2010)
* N. yunnanensis *	KUMCC 20-0267 (HT)	MW729423	MW717892	MW881141	MW717890	Tibpromma et al. (2021)
* Podosordaria mexicana *	WSP 176	GU324762	GQ853039	GQ844840	GQ455451	Hsieh et al. (2010)
* Pod. muli *	WSP 167 (HT)	GU324761	GQ853038	GQ844839	GQ455450	Hsieh et al. (2010)
* Poronia pileiformis *	WSP 88113001 (ET)	GU324760	GQ853037	GQ502720	GQ455449	Hsieh et al. (2010)
* Por. punctata *	CBS 656.78 (HT)	KT281904	KY624278	KX271281	N/A	Senanayake et al. (2015)
* Rosellinia aquila *	MUCL 51703	KY610392	KY624285	KX271253	N/A	Wendt et al. (2018)
* R. merrillii *	HAST 89112601	GU300071	GQ844781	GQ470229	GQ398229	Hsieh et al. (2010)
* R. sanctae-cruciana *	HAST 90072903	GU292824	GQ844777	GQ470227	GQ389699	Hsieh et al. (2010)
* Stilbohypoxylon elaeicola *	HAST 94082615	GU322440	GQ844827	GQ495933	GQ438754	Hsieh et al. (2010)
* S. quisquiliarum *	HAST 89091608	EF026120	GQ853021	EF025606	EF025591	Ju et al. (2007), Hsieh et al. (2010)
* Xylaria allantoidea *	HAST 94042903	GU324743	GQ848356	GQ502692	GQ452377	Hsieh et al. (2010)
* X. apoda *	HAST 90080804	GU322437	GQ844823	GQ495930	GQ438751	Hsieh et al. (2010)
* X. compunctum *	CBS 359.61	KT281903	KY624230	KX271255	N/A	Senanayake et al. (2015)
* X. cubensis *	JDR 860	GU991523	GQ848365	GQ502700	GQ455444	Hsieh et al. (2010)
* X. digitata *	HAST 919	GU322456	GQ848338	GQ495949	GQ449245	Hsieh et al. (2010)
* X. juruensis *	HAST 92042501	GU322439	GQ844825	GQ495932	GQ438753	Hsieh et al. (2010)

Notes: Type specimens are marked with HT (holotype), ET (epitype). N/A: sequences not available.

### Sequence alignment and phylogenetic analyses

Except for newly-generated sequences, all sequences used for phylogenetic analysis were downloaded from GenBank, based on published literature and the highest hit rate of ITS in the GenBank database. Sequence data for the construction of the phylogenetic tree are listed in Table 1. Sequence alignments were generated using the MAFFT v.7.110 online programme (http://mafft.cbrc.jp/alignment/server/, Katoh and Standley 2013) under default settings. Multiple sequence alignments of ITS, α-actin, *rpb2* and β-tubulin were analysed individually and in combination, manually adjusted to achieve the maximum alignment and to minimise gaps using the BioEdit v.5 (Hall 1999). The file formats were converted in ALTER (Alignment Transformation EnviRonment) (http://www.sing-group.org/ALTER/). The Maximum Likelihood analysis was carried out with GTR+G+I model of site substitution by using RAxML 7.4.2 black box (https://www.phylo.org/, Stamatakis et al. 2008) and Bayesian Inference (BI) analysis was performed with MrBayes v.3.1.2 (Huelsenbeck and Ronquist 2001). The branch support was evaluated with a bootstrapping method of 1000 replicates (Hillis and Bull 1993). Posterior probabilities (PP) were determined by Markov Chain Monte Carlo sampling (MCMC) in MrBayes v. 3.2.2 (Ronquist et al. 2012). The nucleotide substitution model was estimated by MrModeltest v.2.3 (Posada and Crandall 1998). Six simultaneous Markov chains were run for 2000000 generations and the trees were sampled each 100^th^ generation. The first 25% of trees were discarded during the burn-in phase of each analysis. The phylogenetic trees were viewed in Figtree v.1.4.0 and arranged by Photoshop CS6. The alignments and respective phylogenetic trees were uploaded in TreeBASE (submission number: 28371).

## Results

### Phylogenetic analyses

**Figure 1.**
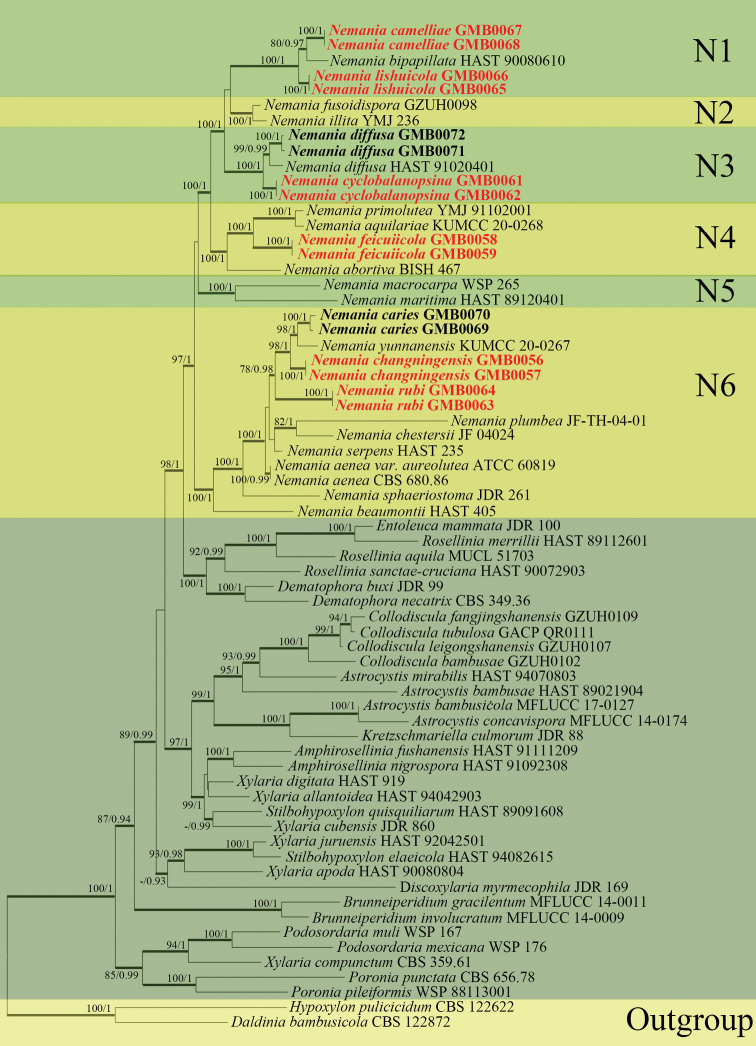
RAxML tree based on analysis of a combined dataset of ITS, α-actin, *rpb2* and β-tubulin sequences from taxa of *Nemania* and related genera. Bayesian posterior probability (PP) ≥ 0.90 is marked at the node and the maximum likelihood bootstrap support (BS) values greater than ≥ 75%; a dash (“-”) indicates a value < 0.90 (PP) or < 75% (BS). The strain number is indicated after the species name. The here-studied strains are in **bold** and new species are indicated in red.

The multiple-genes sequence alignments of ITS, α-actin, *rpb2* and β-tubulin included 67 taxa, 2,041 positions including gaps (ITS: 1–486, α-actin: 487–677, *rpb2*: 678–1,715, β-tubulin: 1,716–2,041). *Daldiniabambusicola* Y.M. Ju et al. (CBS 122872) and *Hypoxylonpulicicidum* J. Fourn. et al. (CBS 122622) were selected as the outgroup taxa. A best-scoring ML tree is represented in Fig. 1. RAxML bootstrap support value ≥ 75% and Bayesian posterior probabilities (BYPP) value ≥ 0.90 are shown above the branches and indicated as thickened lines.

In the phylogenetic tree (Fig. 1), *Nemania* Gray is a sister taxon to the genera *Rosellinia* De Not., *Dematophora* R. Hartig and *Entoleuca* Syd. *Nemania* was divided into six sub-clades. In clade N1, *N.bipapillata* (Berk. & M.A. Curtis) Pouzar, *N.camelliae* sp. nov. and *N.lishuicola* sp. nov. grouped with high statistical values (100/1). In clade N2, *N.fusoidispora* Q.R. Li et al. and *N.illita* (Schwein.) Pouzar. grouped with high statistical values (100/1). Clade N3 contained the frequent species *N.diffusa* (Sowerby) S.F. Gray along with *N.cyclobalanopsina* sp. nov. grouping with high statistical values (100/1). In clade N4, *N.feicuiensis* sp. nov. with *N.abortiva* J.D. Rogers et al., *N.aquilariae* Tibpromma & Lu and *N.primolutea* Y.M. Ju et al. grouped with high statistical values (100/1). Within clade N5, *N.macrocarpa* Y.M. Ju & J.D. Rogers clustered in a well-supported sub-clade with *N.maritima* Y.M. Ju & J.D. Rogers with high statistical values (100/1). Clade N6 comprised *N.changningensis* sp. nov., *N.yunnanensis* Tibpromma & Lu, *N.caries* (Schwein.) Y.M. Ju & J.D. Rogers, *N.rubi* sp. nov., *N.plumbea* A.M.C. Tang et al., *N.chestersii* (J.D. Rogers & Whalley) Pouzar, *N.serpens* (Pers.) Gray with *N.aenea* (Nitschke) Pouzar, N.aeneavar.aureolutea (L.E. Petrini & J.D. Rogers) Y.M. Ju & J.D. Rogers, *N.sphaeriostomum* (Schwein.) Lar.N. Vassiljeva & S.L. Stephenson and *N.beaumontii* (Berk. & M.A. Curtis) Y.M. Ju & J.D. Rogers grouping with high support values (100% ML, 1 BYPP).

### Taxonomy

#### Nemania camelliae

Taxon classificationFungiXylarialesXylariaceae

Y.H. Pi & Q.R. Li sp. nov.

A7174B7E-6E29-5054-B459-F205538E01C5

840086

Fig. 2

##### Etymology.

Refers to the host genus name, *camellia*.

##### Material examined.

China, Guizhou Province, Tongren City, Fanjingshan Nature Reserve (27°47'11.41"N, 108°43'43.90"E, altitude: 515 m), on dead wood of *Camellia* sp., 15 October 2020, Y.H. Pi, 2020FJS26 (GMB0068, ***holotype***; GMBC0068, ex-type living culture; KUN-HKAS 112689, ***isotype***).

**Figure 2.**
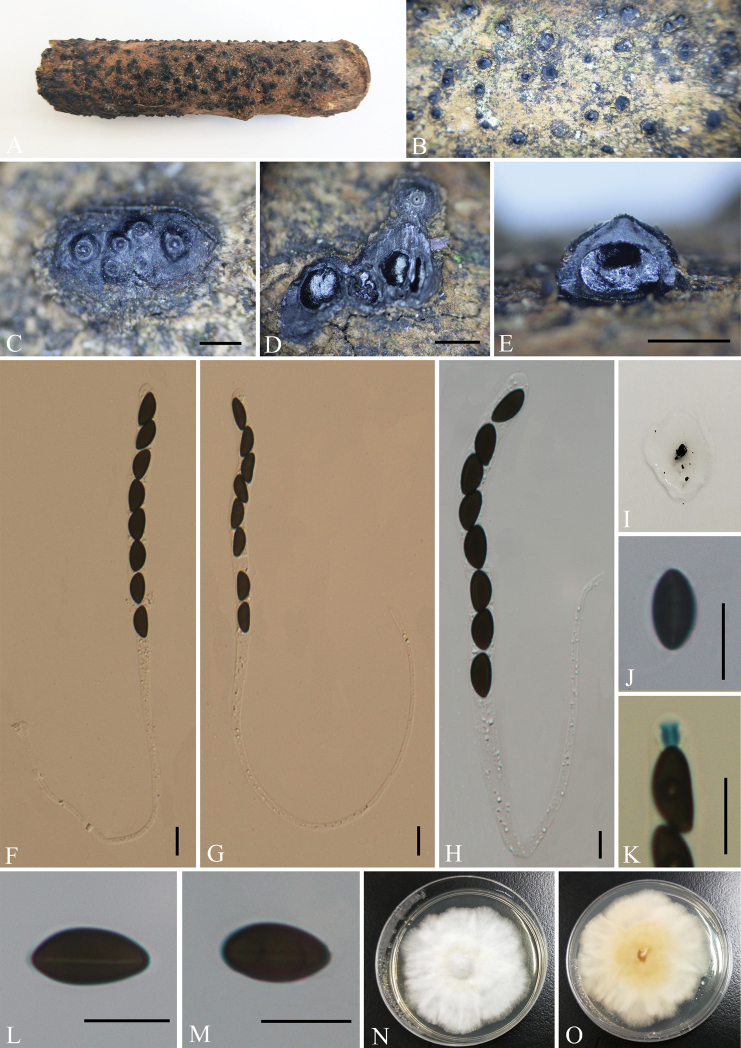
*Nemaniacamelliae* (GMB0068, **holotype**) **A** type material **B, C** stromata on the surface of host **D** transverse section of stroma **E** longitudinal section of stroma **F–H** asci with ascospores **I** pigments in 10% KOH**J** ascospore with indehiscent perispore in 10% KOH**K** ascus apical apparatus (stained in Melzer’s Reagent) **L, M** ascospores **N, O** colonies on PDA (**N**-upper, **O**-lower). Scale bars: 0.5 mm (**C–E**); 10 μm (**F–H, J–M**).

##### Description.

Saprobic on the surface of decaying wood of *Camellia* sp. ***Sexual morph***: Stromata pulvinate to effused-pulvinate, rarely perithecioid, orbicular to irregularly elongated, often coalescent; single distribution or confluent into irregularly elongated compound stromata, 1.5–4 mm long × 1–2 mm wide × 0.5–1 mm high, surface dull black, hard-textured, with inconspicuous to moderately exposed perithecial contours and usually sloping margins, internally black between ascomata, carbonaceous; subperithecial tissue black, conspicuous; does not release a coloured pigment in 10% KOH. Perithecia 0.65–0.95 mm diam. × 0.65–0.7 mm high, subglobose to depressed-spherical. Ostioles finely papillate, black, conspicuously sunken in a shallow discoid depression; ostiolar area blackish, shiny, frequently flattened. Asci 180–290 × 6–11 μm (av. = 230 × 7.5 μm, n = 30), 8-spored, unitunicate, long-cylindrical, long-stipitate, the spore-bearing parts 80–95 µm long, apically rounded with a J+, apical apparatus, 2–3 × 2.5–4 µm (av. = 2.5 × 3 µm, n = 30), jar shape. Ascospores 10–14 × 4.5–7 μm (av. = 12 × 5.5 μm, n = 30), uniseriate, unicellular, ellipsoid to slightly fusoid, inequilateral, with slightly narrow rounded ends, smooth, brown to dark brown, with a fairly conspicuous, straight, almost spore-length germ slit on the least convex side; lacking a sheath and appendage; perispore indehiscent in 10% KOH. ***Asexual morph***: Undetermined.

##### Culture characteristics.

The colony grows on PDA medium with a diameter of 6 cm after one week at 25 °C; white, cottony, circular, flocculent or velvety, with light yellow to slightly yellow at the centre. Not sporulating on OA nor on PDA.

##### Other examined material.

CHINA, Guizhou Province, Tongren City, Fanjingshan Nature Reserve (27°42'10.26"N, 108°31'35.34"E, altitude: 426 m), on dead wood of *Camellia* sp., 16 October 2020, Y.H. Pi, 2020FJS54-1 (GMB0067), living culture, GMBC0067.

##### Notes.

Phylogenetic analyses showed that *Nemaniacamelliae* form a distinct clade with *N.bipapillata* (82% ML, 0.97 BYPP, Fig. 1). Morphologically, *N.camelliae* is similar to *N.immersidiscus* Van der Gucht et al. in having a small discoid depression around the ostiolar papilla. However, the stromata of *N.camelliae* are entirely carbonaceous, whereas those of *N.immersidiscus* contain white soft tissue between and beneath the perithecia (Ju and Rogers 2002). Moreover, *N.immersidiscus* has slightly thinner ascospores [(10–)11–14(–16) × (4–)4.5–5.5 µm)].

#### Nemania caries

Taxon classificationFungiXylarialesXylariaceae

(Schwein.) Y.M. Ju & J.D. Rogers, Nova Hedwigia 74(1–2): 90 (2002)

2942CB75-0976-5BB2-BF37-7EDB687B42D5

477305

Fig. 3

Synonyms. Sphaeriacaries Schwein., Trans. Am. phil. Soc., New Series 4(2): 194 (1832).
Hypoxylon caries (Schwein.) Sacc., Syll. fung. (Abellini) 1: 393 (1882).
Hypoxylon balansae Speg., Anal. Soc. cient. argent. 26(1): 30 (1888).

##### Description.

Saprobic on the surface of decaying wood. ***Sexual morph***: Stromata irregularly effused-pulvinate, 5.5–18 mm long × 3–9 mm wide × 0.4–0.6 mm thick, with conspicuous perithecial mounds, surface blackish-grey, carbonaceous, interior white, loosely fibrous to cottony; mature stromata lacking KOH extractable pigments. Perithecia 0.25–0.5 mm wide × 0.4–0.6 mm high, obovoid. Ostioles slightly higher than stromatal surface and with openings conic-papillate, black, inconspicuous, without encircling disc. Asci 130–200 × 7–13 μm (av. = 150 × 9.5 μm, n = 30), 8-spored, cylindrical, unitunicate, long-stipitate, the spore-bearing parts 65–95 µm long, apically rounded with a J+, short-cylindrical apical apparatus, 1.5–2.5 × 1–2.5 µm (av. = 2 × 1.5 µm, n = 30). Ascospores 9–13.5 × 3–7 μm (av. = 11.5 × 5 μm, n = 30), brown to light brown, smooth, with an inconspicuous, straight, germ slit 1/3 spore-length, nearly equilateral, with broadly rounded ends; perispore indehiscent in 10% KOH. ***Asexual morph***: Undetermined.

**Figure 3.**
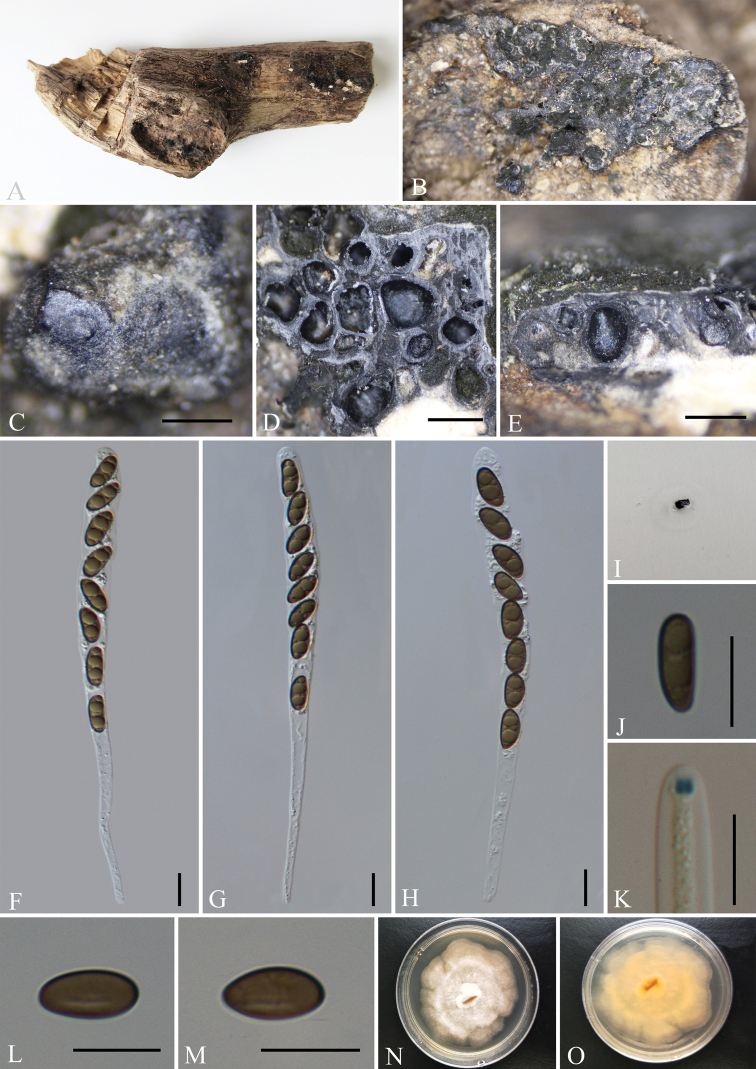
*Nemaniacaries* (GMB0070) **A** type material **B, C** stromata on the surface of host **D** transverse sections of stromata **E** longitudinal section of stroma **F–H** asci with ascospores **I** pigments in 10% KOH**J** ascospore with indehiscent perispore in 10% KOH**K** ascus apical apparatus (stained in Melzer’s Reagent) **L, M** ascospores **N, O** Colonies on PDA (**N**-upper, **O**-lower). Scale bars: 0.5 mm (**C–E**); 10 μm (**F–H, J–M**).

##### Culture characteristics.

Colonies grow on PDA at 25 °C for two weeks, with a diameter of 4 cm. Colony on the surface is white or light orange, shallow, flat, zonnate, with irregular edges and orange on the reverse side. The colony reverse is orange. Not sporulating on OA nor on PDA.

##### Material examined.

China, Yunnan Province, Changning County, Lancang River Nature Reserve (25°01'13.56"N, 99°35'25.12"E, altitude: 2626 m), on dead wood, 6 October 2019, Y.H. Pi, 2019LC369 (GMB0070, KUN-HKAS 112680), living culture, GMBC0070; CHINA, Yunnan Province, Changning County, Lancang River Nature Reserve (25°01'13.33"N, 99°35'26.55"E, altitude: 2641 m), on dead wood, 6 October 2019, Y.H. Pi, 2019LC401 (GMB0069, KUN-HKAS 112682), living culture, GMBC0069.

##### Known distribution.

Hawaii (Rogers and Ju 2012), Martinique (Fournier et al. 2018), Paraguay, USA (Ju and Rogers 2002), Yunnan Province, China (this paper).

##### Notes.

The phylogenetic analyses show *Nemaniacaries* groups with *N.changningensis* with high statistical support (100% ML, 1 BYPP, Fig. 1) and the comparison calculation within the alignment found that there is a 4% difference in ITS sequences between *N.changningensis* and *N.caries*. Morphologically, *N.caries* resembles *N.colubrina* J. Fourn. & Lechat which has medium brown ascospores and a similar size of ascospores. However, *N.colubrina* differs from *N.caries* by ellipsoid-inequilateral ascospores with narrowly-rounded ends (Ju and Rogers 2002; Fournier et al. 2018). *Nemaniacaries* is distinguished from *N.plumbea* by its dimension of ascospores, the latter has larger ascospores (13–16 × 5.4–6.6 µm) with narrowly-rounded ends (Tang et al. 2007). The specimens we collected from the Lancang River Nature Reserve in Yunnan fit the definition of *N.caries* well and represent the first record from China.

#### Nemania changningensis

Taxon classificationFungiXylarialesXylariaceae

Y.H. Pi & Q.R. Li sp. nov.

B12D7ACE-13B0-50F2-99AA-4AE593E98ED3

840087

Fig. 4

##### Etymology.

Refers to the collection location, Changning County.

##### Material examined.

China, Yunnan Province, Changning County, Lancang River Nature Reserve (25°01'35.02"N, 99°33'15.42"E, altitude: 2670 m), on dead wood, 3 October 2019, Y.H. Pi, 2019LC203 (GMB0056, ***holotype***; GMBC0056, ex-type living culture; KUN-HKAS 112668, ***isotype***).

##### Description.

Saprobic on the surface of decaying wood. ***Sexual morph***: Stromata effused-pulvinate, confluent into irregularly elongated compound stromata, up to 18–35 mm long × 2–4 mm wide × 0.3–0.5 mm high, irregularly lobed, plane or with inconspicuous perithecial mounds and sloping margins; surface covered with white tissue, persistent layer, with blackish-grey carbonaceous sub-surface showing through in places; the tissue beneath the perithecial layer inconspicuous, greyish-white in places, the underlying wood blackened; mature stromata lacking KOH extractable pigments. Perithecia 0.45–0.6 mm diam. × 0.4–0.55 mm high, subglobose to depressed-spherical. Ostioles slightly higher than stromatal surface and with openings papillate, often surrounded by white tissue, inconspicuous, black, without encircling disc. Asci 100–140 × 7–10 μm (av. = 111 × 8.5 μm, n = 30), 8-spored, unitunicate, cylindrical, short-stipitate, the spore-bearing parts 70–90 µm long, the apical apparatus of immature asci blue in Melzer’s Reagent, but not blue in mature asci. Ascospores 10–13 × 4–6.5 μm (av. = 11.5 × 5.5 μm, n = 30), uniseriate unicellular, smooth, light brown, slightly inequilateral, with broadly rounded ends, inconspicuous or lack a germ slit; perispore indehiscent in 10% KOH. ***Asexual morph***: Undetermined.

**Figure 4.**
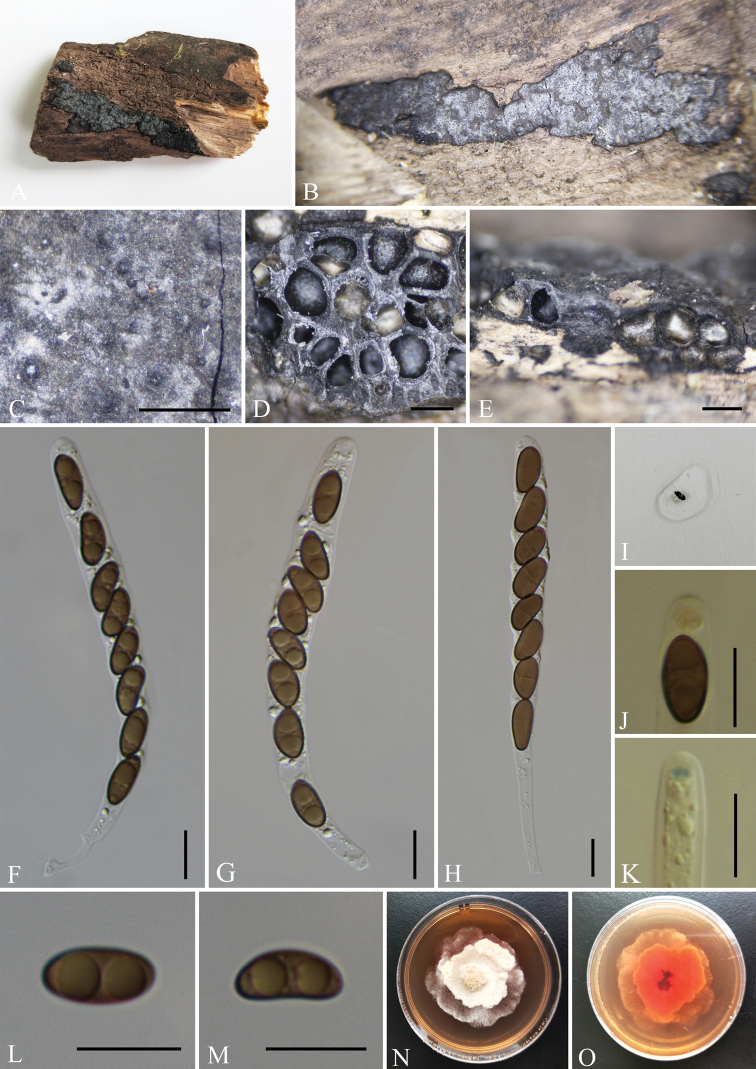
*Nemaniachangningensis* (GMB0056, **holotype**) **A** type material **B, C** stromata on the surface of host **D** transverse sections of stromata **E** longitudinal section of stroma **F–H** asci with ascospores **I** pigments in 10% KOH**J, K** asci apical apparatus (stained in Melzer’s Reagent) **L, M** ascospores **N, O** colonies on PDA (**N**-upper, **O**-lower). Scale bars: 0.5 mm (**C–E**); 10 μm (**F–H, J–M**).

##### Culture characteristics.

The colony grows slowly on the PDA with a diameter of 4.5 cm after 2 weeks at 25 °C. The colony on the surface is white, thick and flat in the middle, edges are shallow, irregular bands and rosettes. Colony reverse is orange and intermediate colour darker. Not sporulating on OA nor on PDA.

##### Other examined material.

China, Yunnan Province, Changning County, Lancang River Nature Reserve (25°01'30.36"N, 99°35'30.53"E, altitude: 2586 m), on dead wood, 4 October 2019, Y.H. Pi, 2019LC342 (GMB0057), living culture, GMBC0057.

##### Notes.

In the phylogenetic analyses, *N.changningensis* is on a separate branch and grouped with *N.caries* with high support values (100% ML, 1 BYPP, Fig. 1). In term of ascospores dimension, *N.changningensis* resembles *N.caries*, but differs in the perithecia of *N.caries* (obovoid, 0.3–0.6 mm diam. × 0.5–0.7 mm high), in the surface not covered with white tissue and in its apical apparatus of mature asci bluing in Melzer’s Reagent (Miller 1961; Ju and Rogers 2002).

#### Nemania cyclobalanopsina

Taxon classificationFungiXylarialesXylariaceae

Y.H. Pi & Q.R. Li sp. nov.

EF877721-557A-583D-B6E3-61998AEF6E01

840088

Fig. 5

##### Etymology.

Refers to its host, *Cyclobalanopsisglauca*.

##### Material examined.

China, Yunnan Province, Changning County, Lancang River Nature Reserve (25°01'9.46"N, 99°35'29.47"E, altitude: 2623 m), on dead wood of *C.glauca*, 6 October 2019, Y.H. Pi, 2019LC357 (GMB0062, holotype; GMBC0062, ex-type living culture; KUN-HKAS 112679, isotype).

##### Description.

Saprobic on the surface of decaying branches of *C.glauca* (Thunb.) Oerst. ***Sexual morph***: Stromata effused-pulvinate, orbicular to ellipsoid or irregularly lobed, 6–26 mm long × 3.5–10 mm wide × 0.5–1 mm thick, occasionally confluent into larger compound stromata, with steep to sloping margins; surface light blackish, slightly blood colour; outer crust carbonaceous; interior black, entire tissue carbonaceous around the perithecia; mature stromata lacking KOH-extractable pigments. Perithecia 0.2–0.3 mm diam. × 0.38–0.46 mm high, subglobose obovoid or tubular. Ostioles higher than stromatal surface and with coarsely rounded-papillate, black, without encircling disc. Asci 90–160 × 7–11 μm (av. = 125 × 9 μm, n = 30), 8-spored, unitunicate, cylindrical, long-stipitate, the spore-bearing parts 65–85 µm long, apically rounded with a J+, short-cylindrical to slightly tubular apical apparatus stained in Melzer’s Reagent, 1.5–2.5 × 2–3 µm (av. = 2 × 2.3 µm, n = 30). Ascospores 9–14 × 4.5–7.5 μm (av. = 11 × 6 μm, n = 30), uniseriate, unicellular, ellipsoid-inequilateral with broadly rounded ends, smooth, brown to dark brown, with a conspicuous, straight germ slit slightly less than spore-length to almost spore-length on the convex side; lacking a sheath and appendage; perispore indehiscent in 10% KOH. ***Asexual morph***: Undetermined.

**Figure 5.**
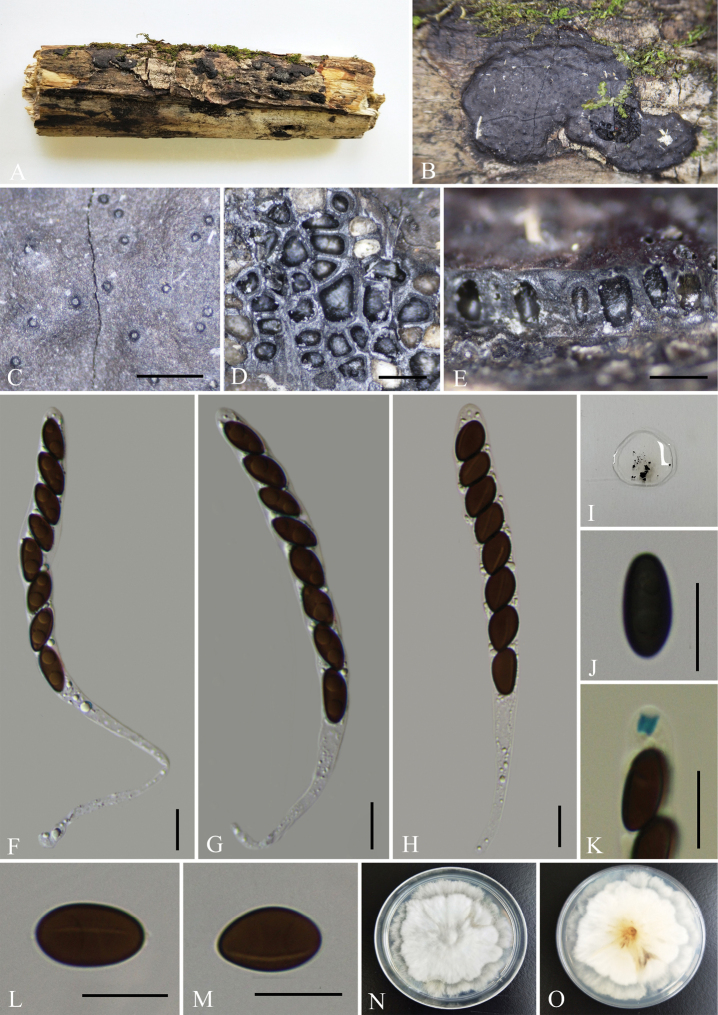
*Nemaniacyclobalanopsina* (GMB0062, **holotype**) **A** type material **B, C** stromata on the surface of host **D** transverse sections of stromata **E** longitudinal sections of stromata **F–H** asci with ascospores **I** pigments in 10% KOH**J** ascospore with indehiscent perispore in 10% KOH**K** ascus apical apparatus (stained in Melzer’s Reagent) **L, M** ascospores **N, O** colonies on PDA (**N**-upper, **O**-lower). Scale bars: 0.5 mm (**C–E**); 10 μm (**F–H, J–M**).

##### Culture characteristics.

Colonies on PDA medium in size with a diameter of 6 cm after two weeks at 25 °C; the surface is white, intermediate thick, cottony, dense, with undulate or ring edge, flat, low, whitish-yellow, reverse of the colony yellow at the centre. Not sporulating on OA nor on PDA.

##### Other examined material.

China, Yunnan Province, Changning County, Lancang River Nature Reserve (25°52'17.40"N, 99°35'20.53"E, altitude: 1489 m), on dead wood of *C.glauca*, 4 October 2019, Y.H. Pi, 2019LC357-1 (GMB0061), living culture, GMBC0061.

##### Notes.

In our phylogenetic analyses, *N.cyclobalanopsina* grouped with *N.diffusa* (100% ML, 1 BYPP, Fig. 1). Morphologically, *N.cyclobalanopsina* differs from *N.diffusa* by its blackish stromatal surfaces and coarsely rounded-papillate ostioles. Moreover, *N.diffusa* has larger perithecia (0.3–0.6 × 0.4–0.8 mm) (Granmo et al. 1999; Ju and Rogers 2002). In the multi-gene phylogenetic analysis, *N.cyclobalanopsina* appeared in a separate branch which is distinct from *N.diffusa* (Fig. 1). Moreover, there is a 3% difference in ITS sequences between *N.diffusa* and *N.cyclobalanopsina*. (Vu et al. 2019; Jeewon and Hyde 2016).

#### Nemania diffusa

Taxon classificationFungiXylarialesXylariaceae

(Sowerby) S.F. Gray, Nat. Arr. Brit. Pl.: 517 (1821)

7979F807-0FA7-5A95-B671-3796EC6D104C

477312

Fig. 6

Synonyms. Sphaeriadiffusa Sowerby, Col. fig. Engl. Fung. Mushr. (London) 3(no. 25): tab. 373, fig. 10 (1802)
Sphaeria unita Fr., Elench. fung. (Greifswald) 2: 67 (1828)
Sphaeria exarata Schwein., Trans. Am. phil. Soc., New Series 4(2): 192 (1832)
Hypoxylon exaratum (Schwein.) Sacc., Syll. fung. (Abellini) 1: 392 (1882)
Ustulina linearis Rehm, Hedwigia 31(6): 310 (1892)
Hypoxylon lilacinofuscum Bres., Fl. Trident. Nov. 2: 43 (1892)
Hypoxylon cohaerens var. brasiliense Starbäck, Bih. K. svenska VetenskAkad. Handl., Afd. 3 27(no. 9): 8 (1901)
Hypoxylon vestitum Petch, Ann. R. bot. Gdns Peradeniya 8: 156 (1924)
Nemania unita (Fr.) Krieglst. & Enderle, Mitteilungsblatt der Arbeitsgemeinschaft Pilzkunde Niederrhein 1: 64 (1989)

##### Description.

Saprobic on the surface of rotten wood. ***Sexual morph***: Stromata effused-pulvinate, clear outline, ellipsoid or irregularly lobed, occasionally confluent into a larger compound stromata, 2–20 mm long × 2–9 mm wide × 0.5–1 mm thick, with conspicuous perithecial mounds, carbonaceous between the perithecia, surface dark brown or brown; the inter-perithecial tissue blackish, carbonaceous; does not release a coloured pigment in 10% KOH. Perithecia 0.3–0.55 diam. × 0.4–0.7 mm high, subglobose to obovoid. Ostioles finely conic-papillate, black, shiny. Asci 130–250 × 6–10 μm (av. = 170 × 8 μm, n = 30), 8-spored, unitunicate, cylindrical, long-stipitate, the spore-bearing parts 70–90 µm, apically rounded with a J+ apical apparatus, 1.5–2.5 × 2–3.5 µm (av. = 2 × 2.6 µm, n = 30), tubular with a faint upper rim, bluing in Melzer’s Reagent. Ascospores 9.5–13 × 4.5–7 μm (av. = 11 × 5.5 μm, n = 30), unicellular, ellipsoid-inequilateral, with narrowly-rounded ends, smooth, brown to dark brown, with a conspicuous, straight germ slit spore-length to slightly less than spore-length on the ventral side; lacking a sheath and appendage; perispore indehiscent in 10% KOH. ***Asexual morph***: Undetermined.

**Figure 6.**
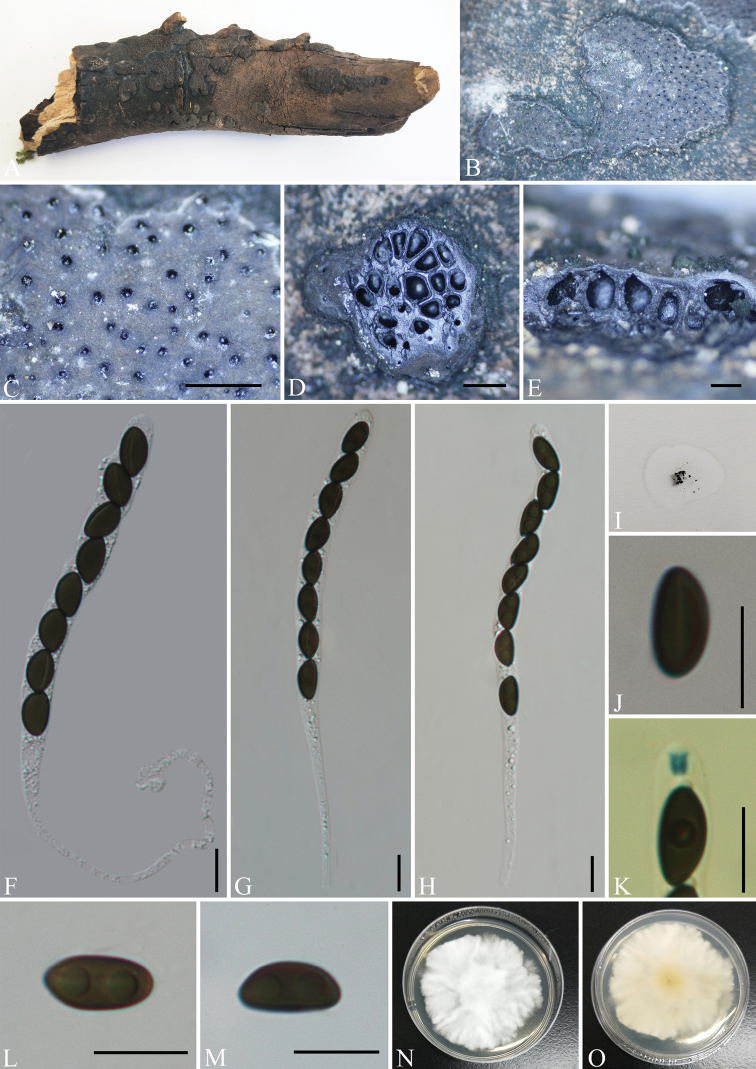
*Nemaniadiffusa* (GMB0072) **A** specimen **B, C** stromata on the surface of host **D** transverse sections of stromata **E** longitudinal sections of stromata **F–H** asci with ascospores **I** pigments in 10% KOH**J** ascospore with indehiscent perispore in 10% KOH**K** ascus apical apparatus (stained in Melzer’s Reagent) **L, M** ascospores **N, O** colonies on PDA (**N**-upper, **O**-lower). Scale bars: 0.5 mm (**C–E**); 10 μm (**F–H, J–M**).

##### Culture characteristics.

Colonies grow on PDA at 25 °C for a week reaching a diameter of 5 cm. Colonies are cotton white in colour, flocculent or velvety, dense, circular, radial. On the reverse, white edge, light yellow in the middle. Not sporulating on OA nor on PDA.

##### Material examined.

China, Guizhou Province, Tongren City, Fanjingshan Nature Reserve (27°53'46.59"N, 108°431'16.29"E, altitude: 1058 m), on dead wood, 14 October 2020, Y.H. Pi, 2020FJS1 (GMB0072, KUN-HKAS 112686), living culture, GMBC0072; CHINA, Yunnan Province, Changning County: Lancang River Nature Reserve (21°54'17.44"N, 107°54'10.05"E, altitude: 1382 m), on dead wood, 1 October 2019, Y.H. Pi, 2019LC008 (GMB0071, KUN-HKAS 112658), living culture, GMBC0071.

##### Notes.

The new collection morphologically resembles *N.diffusa* (Gray 1821), having effused-pulvinate carbonaceous stromata with inconspicuous perithecial mounds, brown to dark brown ellipsoid-inequilateral ascospores (9.5–13.5 × 5–6 µm), with narrowly-rounded ends and a long germ slit on the ventral side (Granmo et al. 1999; Ju and Rogers 2002). Fournier et al. (2018) predicted that *N.diffusa* might be a species complex as it is difficult to identify, based solely on morphology, thus, it should be evaluated after extensive sampling and using DNA-based taxonomy. In phylogenetic analyses of combined ITS, *rpb2*, β-tubulin and α-actin genes (Fig. 1), new collections clearly showed its close kinship with *N.diffusa*. Only a 2% difference of ITS sequences existed between our strains and *N.diffusa* (HAST 91020401, authoritative strain). Therefore, we regard the new collection as *N.diffusa*. *Nemaniacarbonacea* Pouzar. can be confused with *N.diffusa* by having the same dark ascospores and nearly spore-length germ slits. However, *N.carbonacea* has white, soft stromatal tissue between the perithecia (Ju and Rogers 2002).

#### Nemania feicuiensis

Taxon classificationFungiXylarialesXylariaceae

Y.H. Pi & Q.R. Li sp. nov.

464D8645-2CB7-5AD4-8926-6AE4B3429715

840089

Fig. 7

##### Etymology.

Refers to the collection location, Emerald Park, Chinese name of jade, feicui.

##### Material examined.

China, Hainan Province, Wuzhishan City, Emerald Park (18°48'9.64"N, 109°31'6.59"E, altitude: 352 m), on dead wood, 14 November 2020, Y.H. Pi, 2020FCGY12-2 (GMB0059, ***holotype***; GMBC0059, ex-type living culture; KUN-HKAS 112698, ***isotype***).

##### Description.

Saprobic on the surface of decaying wood. ***Sexual morph***: Stromata effused-pulvinate, superficial, orbicular to ellipsoid or irregularly lobed, 5–27 mm long × 2.5–10 mm wide × 0.3–0.5 mm thick, surface blackish-grey, with inconspicuous perithecial outer mounds, crust weakly carbonaceous; interior black, stromatal tissue between the perithecia carbonaceous; mature stromata lacking KOH extractable pigments. Perithecia 0.3–0.55 mm diam. × 0.25–0.37 mm high, subglobose to depressed-spherical. Ostioles higher than stromatal surface and with openings slightly papillate, black, conspicuous, without encircling disc. Asci 130–180 × 7–11.5 μm (av. = 145 × 9 μm, n = 30), 8-spored, unitunicate, cylindrical, long-stipitate, the spore-bearing parts 65–85 µm long, apically rounded with a J+ apical apparatus, 1–2.5 × 2–3 µm (av. = 1.8 × 2.4 µm, n = 30), long-cylindrical. Ascospores 9.5–13 × 4–7.5 μm (av. = 11 × 6 μm, n = 30), uniseriate, unicellular, ellipsoid or slightly inequilateral, with broadly rounded ends, smooth, brown to dark brown, with a conspicuous, straight, almost spore-length germ slit on the flattened side; lacking a sheath and appendage; perispore indehiscent in 10% KOH. ***Asexual morph***: Undetermined.

**Figure 7.**
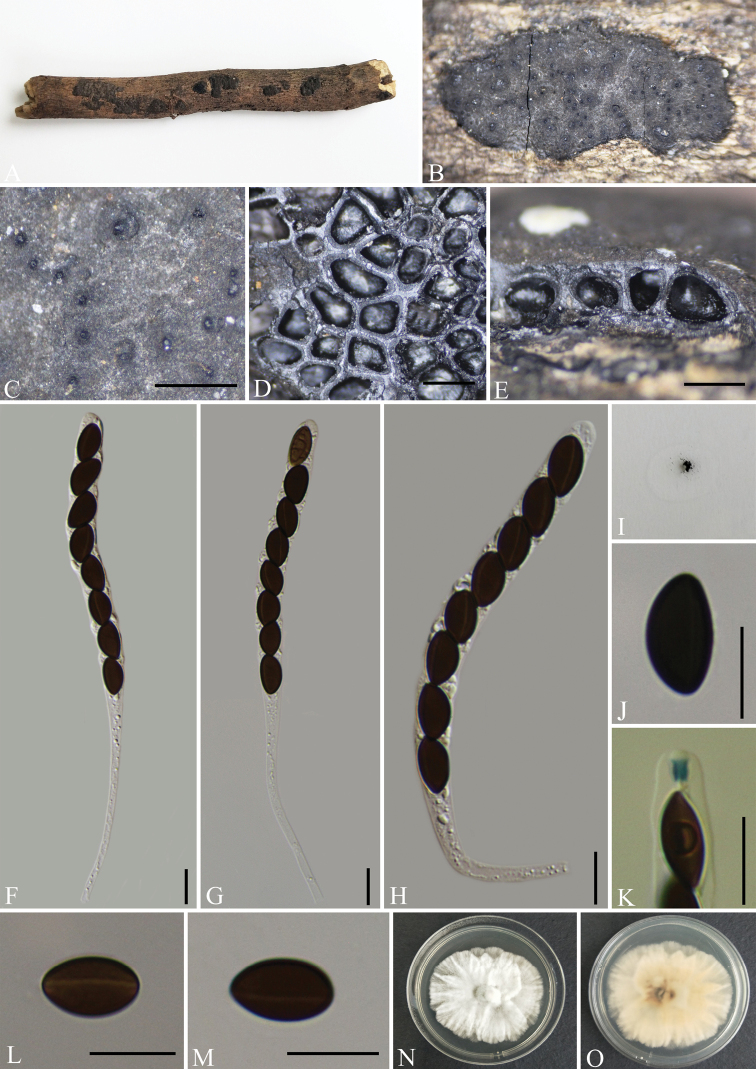
*Nemaniafeicuiensis* (GMB0059, **holotype**) **A** type material **B, C** stromata on the surface of host **D** transverse sections of stromata **E** longitudinal sections of stromata **F–H** asci with ascospores **I** pigments in 10% KOH**J** ascospore with indehiscent perispore in 10% KOH**K** ascus apical apparatus (stained in Melzer’s Reagent) **L, M** ascospores **N, O** colonies on PDA (**N**-upper, **O**-lower). Scale bars: 0.5 mm (**C–E**); 10 μm (**F–H, J–M**).

##### Culture characteristics.

Colonies grow slowly on PDA at 25 °C for 2 weeks, with a diameter of 5 cm. Colonies are cotton white in colour, flocculent or velvety, slightly convex, circular, shallow edges, radial, white to light yellow on the reverse, light brown in the middle. Not sporulating on OA nor on PDA.

##### Other examined material.

China, Hainan Province, Wuzhishan City, Emerald Park (18°47'8.26"N, 109°31'5.34"E, altitude: 426 m), on dead wood, 16 November 2020, Y.H. Pi, 2020FCGY20 (GMB0058), living culture, GMBC0058.

##### Notes.

The phylogenetic tree (Fig. 1) shows that *N.feicuiensis* and *N.primolutea* are closely related (100% ML, 1 BYPP). In morphology, *N.feicuiensis* differs from *N.primolutea* in that the latter has luteous stromatal surface and slightly smaller ascospores (10–13 × 4.5–5.5 μm) with narrowly-rounded ends (Ju et al. 2005). Furthermore, in the multi-gene phylogenetic analysis, *N.feicuiensis* appeared in a separate branch which is distinct from *N.primolutea* (Fig. 1). *Nemaniafeicuiensis* is similar to *N.diffusa* in stromatal anatomy and ascospores size, but differs by ascospores shape (broadly rounded ends vs. narrowly rounded ends) and the larger perithecia of *N.diffusa* (0.3–0.6 × 0.4–0.8 mm) (Ju and Rogers 2002).

#### Nemania lishuicola

Taxon classificationFungiXylarialesXylariaceae

Y .H. Pi & Q.R. Li sp. nov.

E2164D00-8DD6-5121-858C-7E1F9E6EFFB1

840090

Fig. 8

##### Etymology.

Refer to the host, *quercus*.

##### Material examined.

China, Yunnan Province, Changning County: Lancang River Nature Reserve (25°01'7.93"N, 99°35'30.74"E, altitude: 2629 m), on dead bark of *Quercus* sp., 4 October 2019, Y.H. Pi, 2019LC263 (GMB0065, ***holotype***; GMBC0065, ex-type living culture; KUN-HKAS 112673, ***isotype***).

##### Description.

Saprobic on the surface of decaying wood of *Quercus* sp. ***Sexual morph***: Stromata pulvinate, attached to substrate along entire area of the base, containing one to several perithecia, frequently confluent, 1.5–4 mm long × 1–2 mm wide × 0.5–1 mm thick, with conspicuous perithecial mounds, carbonaceous between the perithecia, surface dull black and slightly shiny at maturity, the inter-perithecial tissue blackish, carbonaceous; not releasing a coloured pigment in 10% KOH. Perithecia 0.7–0.95 mm diam. × 0.65–0.85 mm high, subglobose to depressed-spherical. Ostioles coarsely papillate in discoid areas, ostiolar area blackish, shiny, frequently flattened, usually around a circle of white tissue. Asci 150–300 × 7–12 μm (av. = 200 × 9 μm, n = 30), 8-spored, unitunicate, cylindrical, long-stipitate, spore-bearing parts 95–130 µm long, apically rounded with a J+ apical apparatus, 2–3 × 2–3.5 µm (av. = 2.5 × 3 µm, n = 30), tubular with a faint upper rim. Ascospores 12.5–17 × 5–8.5 μm (av. = 15 × 6.5 μm, n = 30), uniseriate, unicellular, ellipsoid-inequilateral, with broadly rounded ends, smooth, brown to dark brown, with a conspicuous, straight germ slit spore-length to slightly less than spore-length on the flattened side; lacking a sheath and appendage; perispore indehiscent in 10% KOH. ***Asexual morph***: Undetermined.

**Figure 8.**
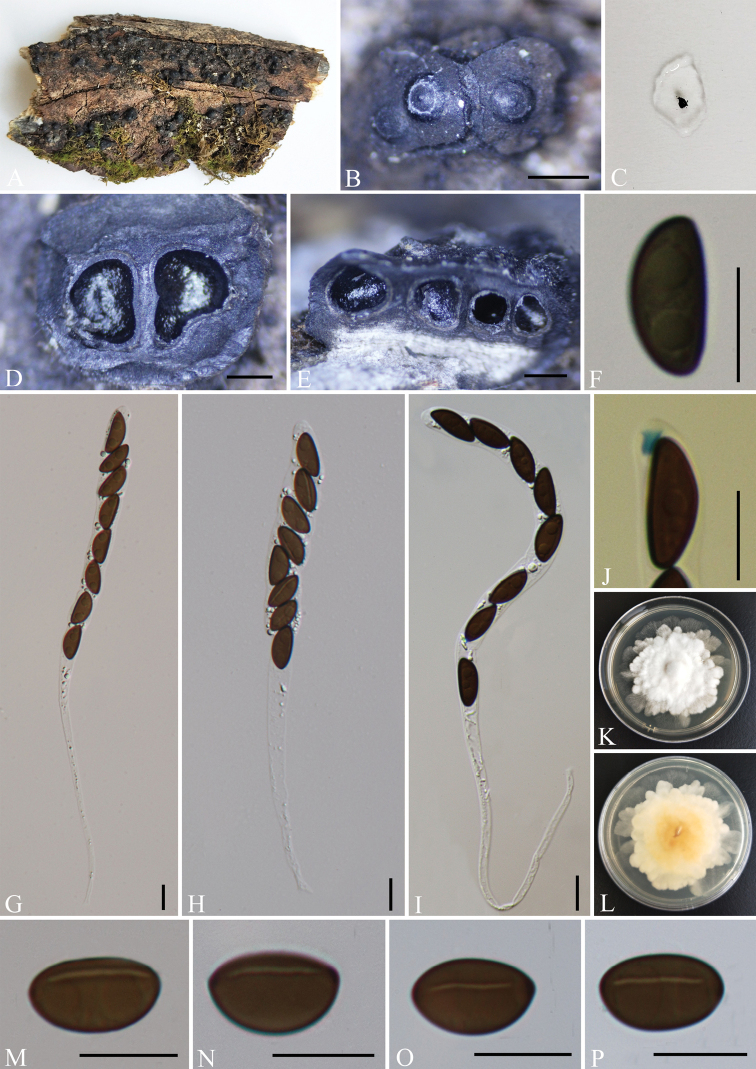
*Nemanialishuicola* (GMB0065, **holotype**) **A** type material **B** stromata on the surface of host **C** pigments in 10% KOH**D** transverse sections of stromata **E** longitudinal sections of stromata **F** ascospore with indehiscent perispore in 10% KOH**G–I** asci with ascospores **J** ascus apical apparatus (stained in Melzer’s Reagent) **K, L** colonies on PDA (**K**-upper, **L**-lower) **M–P** ascospores. Scale bars: 0.5 mm (**B, D, E**); 10 μm (**F–J, M–P**).

##### Culture characteristics.

Colonies grow on PDA, a diameter of 6 cm after one week at 25 °C, white, velvety to hairy, zonnate, rosette, high convex in centre, dense, white to cream from above, white irregular edge with light yellow to slightly yellow at centre from the below. Not sporulating on OA nor on PDA.

##### Other examined material.

China, Yunnan Province, Changning County: Lancang River Nature Reserve (25°01'30.75"N, 99°35'21.53"E, altitude: 2608 m), on dead bark of *Quercus* sp., 4 October 2019, Y.H. Pi, 2019LC253 (GMB0066), living culture, GMBC0066.

##### Notes.

Phylogenetic analyses of combined ITS, *rpb2*, β-tubulin and α-actin genes (Fig. 1) show that *N.lishuicola* has a close relationship with *N.bipapillata* with high support values (100 MLBP, 1% BYPP). Morphologically, *N.lishuicola* differs from *N.bipapillata* by its larger ascospores (12.5–17 × 5–8.5 μm vs. 10.5–13.5 × 4.5–6 μm) (Miller 1961; Ju and Rogers 2002).

#### Nemania rubi

Taxon classificationFungiXylarialesXylariaceae

Y.H. Pi & Q.R. Li sp. nov.

8B64F598-5754-5B0A-A175-D6C55AD5F15E

840091

Fig. 9

##### Etymology.

Refers to the name of host genus, *rubus*.

##### Material examined.

China, Guizhou Province, Pingba County (26°25'13.38"N, 106°24'25.23"E, altitude: 1255 m), on dead branches of *Rubuslambertianus* Ser., 5 September 2020, Y.H. Pi, 2020PB70 (GMB0064, ***holotype***; GMBC0064, ex-type living culture; KUN-HKAS 112695, ***isotype***).

**Figure 9.**
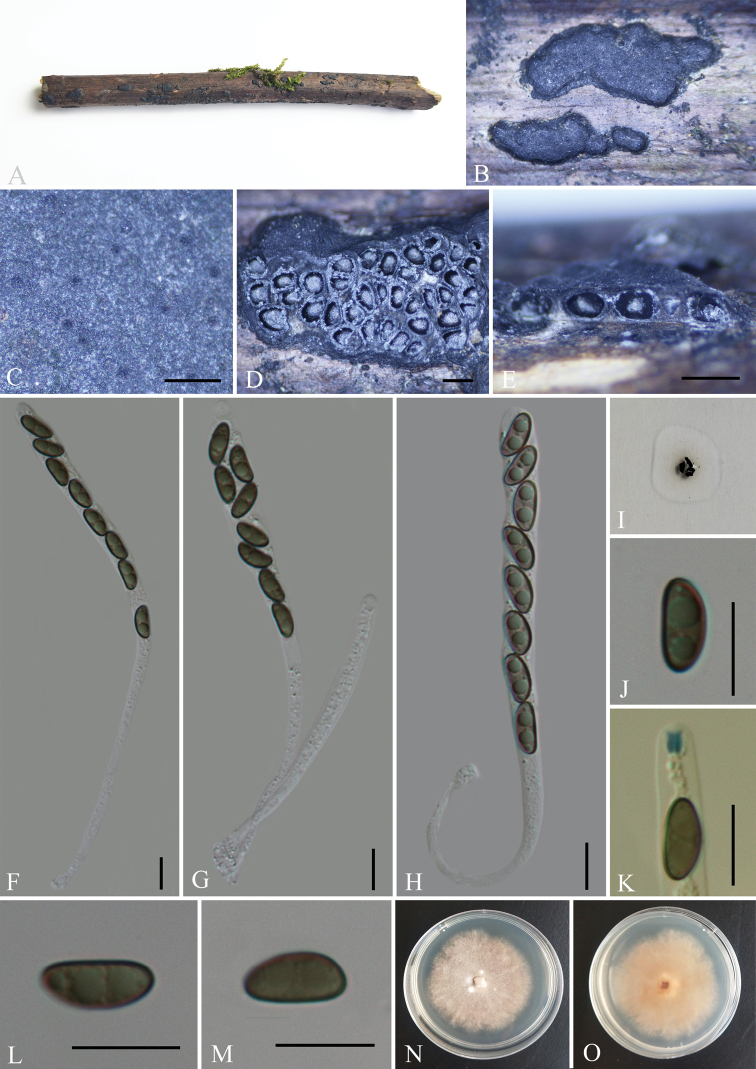
*Nemaniarubi* (GMB0064, **holotype**) **A** type material **B, C** stromata on surface of host **D** transverse sections of stromata **E** longitudinal section of stromata **F–H** asci with ascospores **I** pigments in 10% KOH**J** ascospore with indehiscent perispore in 10% KOH**K** ascus apical apparatus (stained in Melzer’s Reagent) **L, M** ascospores **N, O** colonies on PDA (**N**-upper, **O**-lower). Scale bars: 0.5 mm (**C–E**); 10 μm (**F–H, J–M**).

##### Description.

Saprobic on dead branches of *R.lambertianus*. ***Sexual morph***: Stromata effused-pulvinate, irregular shape, multi-peritheciate, scattered, separate to confluent into larger compound stromata, 2.5–15 mm long × 2–9 mm wide × 0.4–0.6 mm thick; surface blackish, weakly carbonaceous, with unexposed perithecial contours, uneven and irregular, internally whitish between ascomata, tissue, soft-textured; not releasing a coloured pigment in 10% KOH. Perithecia 0.25–0.35 mm diam. × 0.2–0.3 mm high, subglobose. Ostioles papillate, black, obtusely conical to hemispherical, without encircling disc. Asci 85–160 × 7–11 μm (av. = 130 × 9 μm, n = 30), 8-spored, unitunicate, cylindrical, long-stipitate, spore-bearing parts 60–85 µm long, apically rounded with a J+, long-cylindrical apical apparatus, 1.5–2.5 × 2–3 µm (av. = 1.5 × 2.5 µm, n = 30). Ascospores 9–12 × 4–6 μm (av. = 10 × 4.8 μm, n = 30), uniseriate to irregularly-biseriate unicellular, smooth, olivaceous when fresh, turning brown to medium brown after a period of time, ellipsoid-inequilateral with often broadly-rounded ends, lacking a germ slit sheath and appendage; perispore indehiscent in 10% KOH. ***Asexual morph***: Undetermined.

##### Culture characteristics.

Colonies grow slowly on PDA medium with a diameter of 5 cm after 10 days at 25 °C. Colonies surface were white to pale orange, circular, cottony, low, dense, cottony mycelium, reverse with light orange mycelium. Not sporulating on OA nor on PDA.

##### Other examined material.

China, Guizhou Province, Pingba County (26°25'10.24"N, 106°24'25.21"E, altitude: 1052 m), on dead wood, 5 September 2020, Y.H. Pi, 2020PB22 (GMB0063), living culture, GMBC0063.

##### Notes.

In our phylogenetic analysis, *Nemaniarubi* formed a distinct branch, which is sister to *N.changningensis* and *N.caries* (Fig. 1). In morphology, *N.rubi* is similar to *N.caries*, but is distinct in having a long-cylindrical apical apparatus and the inequilateral ascospores lacking a germ slit (Miller 1961; Ju and Rogers 2002). In addition, the perithecia of *N.caries* are obovoid (0.3–0.6 × 0.5–0.7 mm) and its height is greater than the width (Tang et al. 2007). The ascomata surface of *N.rubi* ascomata is uneven with inconspicuous perithecial mounds, which is similar to those of *N.plumbea*, but the latter has larger ascospores (13–16 × 5.4–6.6 µm) with germ slits on the concave side (Tang et al. 2007).

## Discussion

In this study, newly-collected *Nemania* species from Hainan, Yunnan and Guizhou Provinces were subjected to morpho-molecular analyses. Six new species were introduced while reporting one new record from China. *Nemania* showed a closer affinity to *Roselinia* than to *Kretzschmaria* Fr. and *Xylaria* (U’Ren et al. 2016), which is also supported in the phylogenetic analysis, based on ITS, *rpb2*, β-tubulin and α-actin sequences. Although no asexual morphs were observed in this study, *Nemania* has geniculisporium-like asexual morphs which are a common character in members of *Xylariaceae* (Fournier et al. 2018).

*Nemania* forms a single branch in the phylogenetic analysis, which supports that it is a monophyletic genus. However, *Nemania* genus is separated into six clades (N1–N6, Fig. 1), each of which have relatively-uniform morphological characteristics. N1 clade is represented by *N.bipapillata* and taxa in this clade have carbonaceous interior to the stromata, ostioles encircled with a disc and dark brown ascospores with a long germ slit. The species within clade N2 are distinguished from other *Nemania* species with fusoid-inequilateral and pale brown ascospores and by having white soft tissues between the perithecia. The species in clades N3, N4 and N5 have little difference in morphology and may be confused. Most taxa in clades N4 and N5 have usually brown, dark brown or blackish-brown ascospores with a germ slit longer than 2/3 spore length (Granmo et al. 1999; Ju and Rogers 2002; Fournier et al. 2018). The taxa in N6 clade have light brown or medium brown ascospores with a germ slit shorter than 2/3 spore length or seemingly lacking (Ju and Rogers 2002). Interestingly, the ascospores of most taxa in N6 clade are olivaceous brown when fresh, turning medium brown after desiccation.

Separation of members of *Nemania*, based on morphology, is relatively difficult and confusing (Fournier et al. 2018). In some early literature, the new species lacked the description of some key morphological characteristics (Du et al. 2016). Moreover, sequences are available for only a few species in GenBank, thus species identification, based on DNA sequences, is also problematic. Hence, it is essential to re-collect old species that lack ex-type cultures and DNA sequences and to epitypify them.

The similarity of morphological features between species is high, which makes it difficult for existing morphological taxonomic features to identify species. For example, species in clade N3, which includes *N.diffusa* and *N.cyclobalanopsina*, are difficult to identify, based solely on morphological characteristics, although their ITS sequence differences can reach more than 3% (Jeewon and Hyde 2016; Vu et al. 2019). In this clade, we tentatively use multiple-genes sequence as the main classification basis for species. Molecular data should be the main identification basis for *Nemania* species, especially for clade N3. It is worth noting that we should compare sequences with that from type or authoritative strains.

## Supplementary Material

XML Treatment for Nemania camelliae

XML Treatment for Nemania caries

XML Treatment for Nemania changningensis

XML Treatment for Nemania cyclobalanopsina

XML Treatment for Nemania diffusa

XML Treatment for Nemania feicuiensis

XML Treatment for Nemania lishuicola

XML Treatment for Nemania rubi
